# Reconfigurable Magnonic Crystals Based on Imprinted Magnetization Textures in Hard and Soft Dipolar-Coupled Bilayers

**DOI:** 10.1021/acsnano.2c04256

**Published:** 2022-08-31

**Authors:** Krzysztof Szulc, Silvia Tacchi, Aurelio Hierro-Rodríguez, Javier Díaz, Paweł Gruszecki, Piotr Graczyk, Carlos Quirós, Daniel Markó, José Ignacio Martín, María Vélez, David S. Schmool, Giovanni Carlotti, Maciej Krawczyk, Luis Manuel Álvarez-Prado

**Affiliations:** † Institute of Spintronics and Quantum Information, Faculty of Physics, Adam Mickiewicz University, Poznań, Uniwersytetu Poznańskiego 2, 61-614 Poznań, Poland; ‡ Istituto Officina dei Materiali del CNR (CNR-IOM), Sede Secondaria di Perugia, c/o Dipartimento di Fisica e Geologia, Università di Perugia, I-06123 Perugia, Italy; § Departamento de Física, Facultad de Ciencias, Universidad de Oviedo, C/Federico García Lorca no 18, 33007 Oviedo, Spain; ⊥ Centro de Investigación en Nanomateriales y Nanotecnología (CINN), CSIC-Universidad de Oviedo, 33940 El Entrego, Spain; ¶ Institute of Molecular Physics, Polish Academy of Sciences, M. Smoluchowskiego 17, 60-179 Poznań, Poland; ∥ Université Paris-Saclay, UVSQ, CNRS, GEMaC, 78000 Versailles, France; # Dipartimento di Fisica e Geologia, Università di Perugia, I-06123 Perugia, Italy

**Keywords:** spin waves, magnonic crystal, magnetic stripe domains, perpendicular magnetic anisotropy, magnetic bilayers

## Abstract

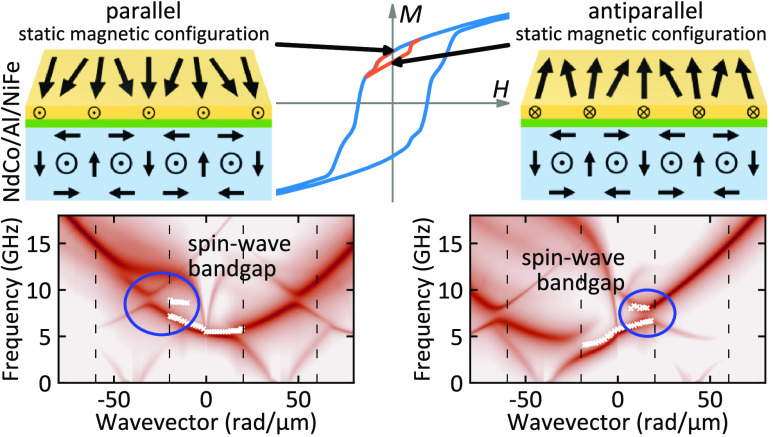

Reconfigurable magnetization textures offer control of spin waves with promising properties for future low-power beyond-CMOS systems. However, materials with perpendicular magnetic anisotropy (PMA) suitable for stable magnetization-texture formation are characterized by high damping, which limits their applicability in magnonic devices. Here, we propose to overcome this limitation by using hybrid structures, i.e., a PMA layer magnetostatically coupled to a low-damping soft ferromagnetic film. We experimentally show that a periodic stripe-domain texture from a PMA layer is imprinted upon the soft layer and induces a nonreciprocal dispersion relation of the spin waves confined to the low-damping film. Moreover, an asymmetric bandgap features the spin-wave band diagram, which is a clear demonstration of collective spin-wave dynamics, a property characteristic for magnonic crystals with broken time-reversal symmetry. The composite character of the hybrid structure allows for stabilization of two magnetic states at remanence, with parallel and antiparallel orientation of net magnetization in hard and soft layers. The states can be switched using a low external magnetic field; therefore, the proposed system obtains an additional functionality of state reconfigurability. This study offers a link between reconfigurable magnetization textures and low-damping spin-wave dynamics, providing an opportunity to create miniaturized, programmable, and energy-efficient signal processing devices operating at high frequencies.

## Introduction

The use of nonuniform magnetic textures to control the propagation of spin waves (SWs) has attracted considerable interest in recent years.^1,2^ This approach has many advantages over conventionally used nanostructured systems at saturation. Their use reduces the negative impact of edge heterogeneity and defects, which can appear in nanofabrication processes. It has been shown that SWs can be guided along domain walls, serving as narrow graded-index waveguides.^3−8^ Domain-wall propagation also removes the limitations related to the bending of SW flow in the in-plane magnetized thin films, existing due to the strong anisotropy of SW dispersion.^6−11^ Particularly interesting in this context are systems with perpendicular magnetic anisotropy (PMA), in which naturally stable Bloch-type domain walls can be very narrow with widths of less than 10 nm, whereas nanostructuring of 50 nm-wide waveguides is still a technological challenge.^12,13^ From an application point of view, the utilization of magnonic circuits with stable magnetic configuration in the absence of the external magnetic field is highly desirable,^14,15^ and one of the best-suited methods to achieve this is to use PMA.^16^ Moreover, it is often possible to achieve various stable magnetic configurations as a medium for the propagation of SWs in the same system, which provides the possibility of reprogrammability.^6,10,17,18^ For instance, in magnetic films with PMA characterized by a quality factor *Q* = 2*K*_PMA_/μ_0_*M*_*S*_^2^ smaller than one (where *K*_PMA_ is the PMA constant and *M*_*S*_ is the saturation magnetization), and above a certain critical thickness, it is possible to stabilize a pattern of aligned stripe domains with alternating up and down out-of-plane magnetization component, having lattice constants of 100 nm and smaller, which can be controlled by the layer thickness.^18−27^ Nevertheless, the thicker the system, the more complex the internal structure of domains and domain walls across the film thickness.^21,28^ However, for Néel- or Bloch-type walls, up and down domains are separated by flux-closure domain walls resembling vortices with cores directed along the domain wall axes.^18,29^ Domain walls in this type of system may also have different chiralities with respect to the polarity of the domain wall that can support nonreciprocal effects for SW propagation.^18,28,29^

Analytical calculations and micromagnetic simulations have indicated that in periodic magnetization textures, the nonlocal dipole field arising from the finite film thickness leads to the formation of a band structure.^30^ More recently, the role of the dipolar field has been also confirmed by Laliena et al.^31^ In this work, the authors have shown theoretically that Bloch domain walls, which are transparent to the SWs propagation when the dipolar interaction is neglected, are able to reflect SWs if the dipolar interaction is taken into account properly.

Periodic stripe domains has been shown to be suitable to control SW dynamics and function as magnonic crystals.^18,28,32−35^ Hitherto, the band-gap openings have only been demonstrated theoretically arising from small lattice constants causing the Brillouin-zone (BZ) edge to lie beyond the experimentally measurable range of wavevectors using conventional techniques. However, the magnonic advantages of thin ferromagnetic films with PMA are limited by the high SW damping usually present in this class of materials. There are a few exceptions to this, such as ferrimagnetic materials with very low saturation magnetization, e.g., bismuth-doped yttrium–iron garnet or thulium–iron garnet.^36−38^

Here we propose an approach, which enables us to avoid high damping of PMA materials while still harnessing the advantages of the magnetization texture for the efficient guiding and control of the SW propagation. For this purpose, we use a hybrid system consisting of a soft ferromagnetic film, which is dipolar-coupled to a hard layer exhibiting a stripe-domain pattern.^39−41^ In previous studies, it has been shown that in this kind of hybrid system, the stripe-domain pattern from the hard magnetic layer is transferred to the soft one.^40,42−45^ Interestingly, in the bilayer system with one layer possessing an out-of-plane component of the static magnetization, the static stray field affects both static and dynamic properties of the second medium.^46^ With this approach, we aim to combine the advantages of regular magnetization textures in PMA films with low-damping thin films, which can be used as an effective low-damping conduit of SWs to advance magnonics.

Moreover, in dipolar-coupled magnetic bilayers, one can achieve nonreciprocal SW propagation so that, for a given frequency, oppositely propagating SWs have different wavelengths.^47−50^ Furthermore, the alternation of the SW amplitude between the layers can be achieved, enabling one to design co- and contra-directional couplers^51^ or utilize the effects to design magnonic diodes and circulators.^52^

In this work, we perform a combined experimental and theoretical study of SW dynamics in a trilayer consisting of a permalloy (Py) film coupled through an Al interlayer (of varying thicknesses) to a NdCo_7.5_ layer with weak PMA, with a ground-state periodic stripe-domain pattern. The static properties of the system have been measured using vibrating sample magnetometry (VSM) and magnetic force microscopy (MFM), whereas SW dynamics have been measured by Brillouin light scattering (BLS) spectroscopy. The experimental results are reproduced and interpreted using finite-element method simulations, which have been further developed to treat the nonuniform magnetic textures. Two different configurations have been stabilized in remanence, and it is possible to switch between them by applying a small external magnetic field: (i) in the parallel state, the magnetization in the Py layer follows the magnetic configuration of the NdCo stripe domains, while (ii) in the antiparallel state, the in-plane magnetization component along the Py stripes axis is antiparallel to that of the NdCo stripes.

The SW dispersion in the Py film is found to be strongly affected by the periodic pattern of the stripe domains in both the parallel and the antiparallel state. In particular, the band structure is characterized by a significant frequency asymmetry induced by the dipolar coupling between the Py and NdCo layer, which becomes more marked with a reduction of the thickness of the Al layer. Moreover, in the sample with the thinnest Al spacer, the opening of a band gap, induced by the hybridization between the fundamental mode in Py from neighboring BZs, has been experimentally observed at the boundary of the first BZ. Interestingly, due to the strong frequency asymmetry of the band structure, the band gap is shifted from the edge of the BZ and occurs only on one side of the experimental dispersion, and is on opposite sides for the parallel and antiparallel state.

## Results and Discussion

### Static Magnetic Properties

**Figure 1:**
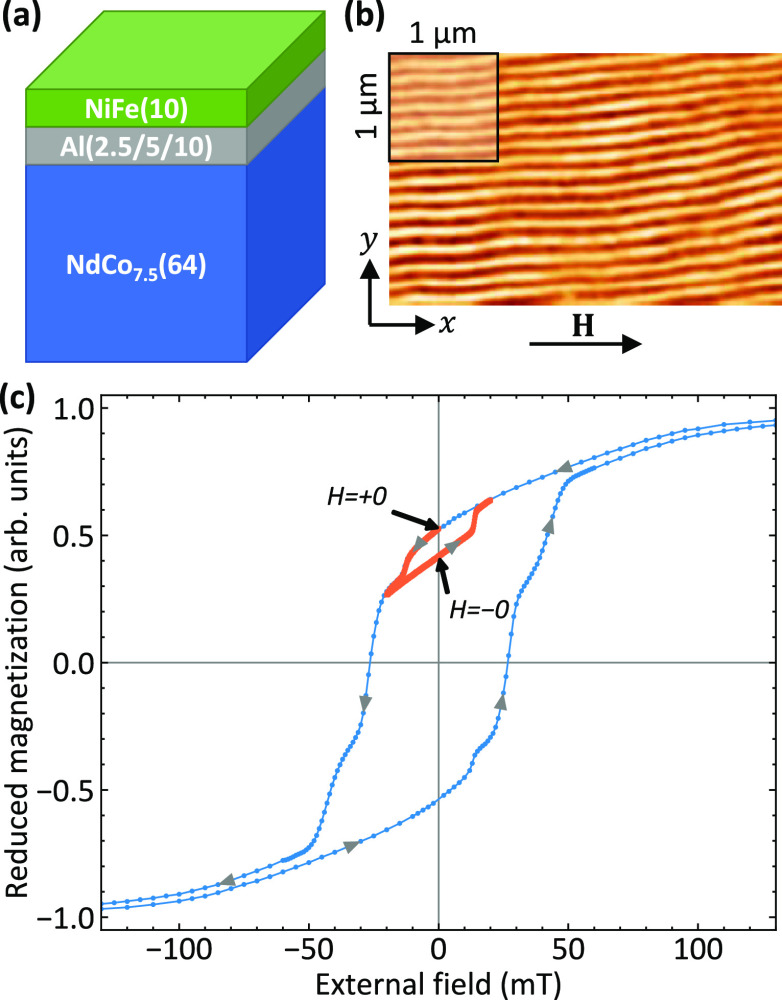
(a) Sketch of the investigated samples. (b) MFM image of the stripe domains taken at remanence after saturation along the *x*-direction for the sample with the 10 nm-thick Al layer. (c) VSM hysteresis loop measured for the sample with the 10 nm-thick Al layer (blue curve). The orange curve refers to the minor loop along which BLS measurements have been performed. The point indicated with *H* = +0 (*H* = –0) marks the remanent state with parallel (antiparallel) longitudinal component of the magnetization (*m*_*x*_) in adjacent stripes.

The investigated trilayer samples (Figure 1a) consist of an amorphous 64 nm-thick NdCo_7.5_ film and a 10 nm-thick Py film coupled through an Al spacer of varying thickness (*t* = 2.5, 5, 10 nm). The fabrication of the samples is described in the Experimental Section. The static magnetic properties of the samples have been studied by means of VSM. Figure 1c shows the hysteresis loop measured by applying a magnetic field along the in-plane easy direction (*x*-axis) for the sample with a 10 nm-thick Al spacer. Similar hysteresis loops have been measured for the other samples. As can be seen, coming from positive saturation, the magnetization curve exhibits the typical “transcritical shape”, characterized by the presence of a linear reversible region starting around 120 mT, corresponding to the formation of stripe domains. When the magnetic field is reversed, a small drop in magnetization, associated with the reversal of the Py magnetization component parallel to the external field, occurs at about −15 mT. Then a plateau is observed until about −25 mT, where the Py and NdCo_7.5_ net magnetizations are aligned along opposite directions, realizing an antiparallel ground state of the system. Next, a second drop can be seen and is ascribed to the reversal of the NdCo_7.5_ magnetization component parallel to the external field. Finally, negative saturation, where stripe domains disappear, is reached at about −120 mT. Furthermore, the minor hysteresis loop (orange curve) has been measured by increasing the magnitude of the applied field in the positive direction once the first plateau, due to the magnetization reversal of Py, has been reached. As can be seen, the state of antiparallel alignment between the Py and the NdCo_7.5_ magnetization remains stable when the magnetic field returns to zero coming from −20 mT (*H* = −0). Moreover, it is possible to recover the parallel alignment of the magnetization in the Py and NdCo layers by applying an in-plane magnetic field *H* = +20 mT and going back to zero (*H* = +0). This shows that we can easily switch between the parallel and antiparallel state by applying a small external magnetic field. The stripe-domain structure was imaged at remanence by MFM. Figure 1b shows an MFM image of the sample with the 10 nm-thick Al spacer. Very regular stripe domains, aligned along the in-plane direction of the last saturating magnetic field, are observed on top surface, i.e., the Py layer, confirming the dipolar interaction between the magnetic layers. From the fast Fourier transform (FFT) of the MFM image, we obtained a stripe domains period (consisting of a couple of ”up” and ”down” domains) of (146 ± 10) nm. A similar stripe-domain pattern has been observed for the other samples (not shown here).

**Figure 2:**
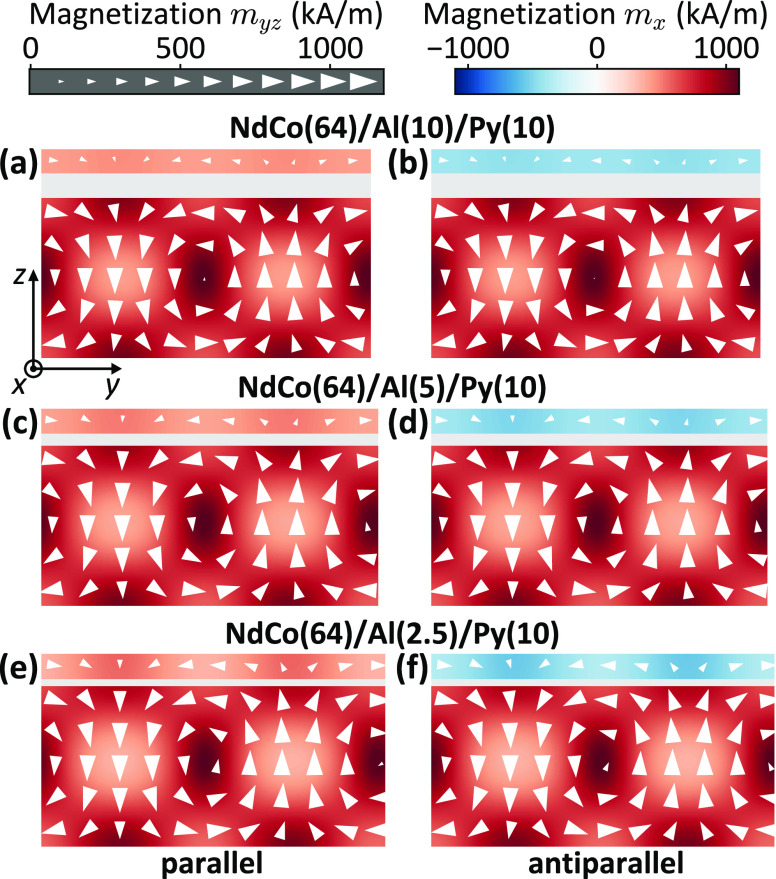
Equilibrium magnetization state calculated for the single unit cell of the trilayer system at remanence for the parallel (left column) and the antiparallel (right column) alignment of the *x*-component of magnetization in Py and NdCo. The results are shown for the samples with the Al layer thickness of (a, b) 10 nm, (c, d) 5 nm, and (e, f) 2.5 nm. The arrows represent the projection of the magnetization in the *yz*-plane, while the component *m*_*x*_ is given by a color code.

To gain a deeper insight into the magnetization configuration of stripe domains at remanence, numerical simulations (for details see Experimental Section) have been performed to visualize the domain structure corresponding to the parallel (*H* = +0) and antiparallel (*H* = −0) remanent states. For the parallel state (Figure 2, left panels), one can observe a magnetic structure in the NdCo film composed of stripe domains, which are alternately magnetized up and down (along the *z*-axis) with respect to the surface plane, separated by Bloch-type domain walls, with the domain-wall cores in-plane magnetized along the saturation direction (+*x*-direction). Flux-closure domains with the in-plane magnetization along the *y*-axis are found at the film surfaces. Note that the flux-closure domain pattern from the top surface of NdCo is transferred to the soft Py layer whose magnetization follows the magnetic configuration of the NdCo stripe domains.

Interestingly, in the numerical simulations, the period of the stripe-domain pattern is found to only slightly increase when the Al thickness decreases, changing from 132 nm [for Al(10)] to 138 nm [for Al(2.5)] due to the increase of the dipolar interaction. Furthermore, with a reduction of the spacer layer thickness, the enhanced imprint of the domain structure in the Py film causes the domain structure itself to expand along the sample thickness and the domain-wall core to shift up toward the Py layer. This effect can be considered as an increase of the effective thickness of the NdCo layer and becomes more pronounced with an increase of the dipolar coupling between the two ferromagnetic layers. Note that the simultaneous increase of the effective thickness of the NdCo layer and of the stripe-domain period is in agreement with the fact that in low-PMA films, the single-domain width should be equal to the thickness of the layer.^19^

For the antiparallel state, the stripe-domain structure in the NdCo film remains stable, maintaining a period identical to that of the parallel state, while in the Py layer, the in-plane magnetization component *m*_*x*_ is observed to reverse along the −*x*-direction. This magnetization configuration has an antiparallel alignment of the *m*_*x*_ magnetization component in Py and NdCo. As in the parallel configuration, the domain-wall core is observed to shift upward into the Py layer when the thickness of the Al spacer is reduced.

### Spin-Wave Dynamics in the Saturated State

**Figure 3:**
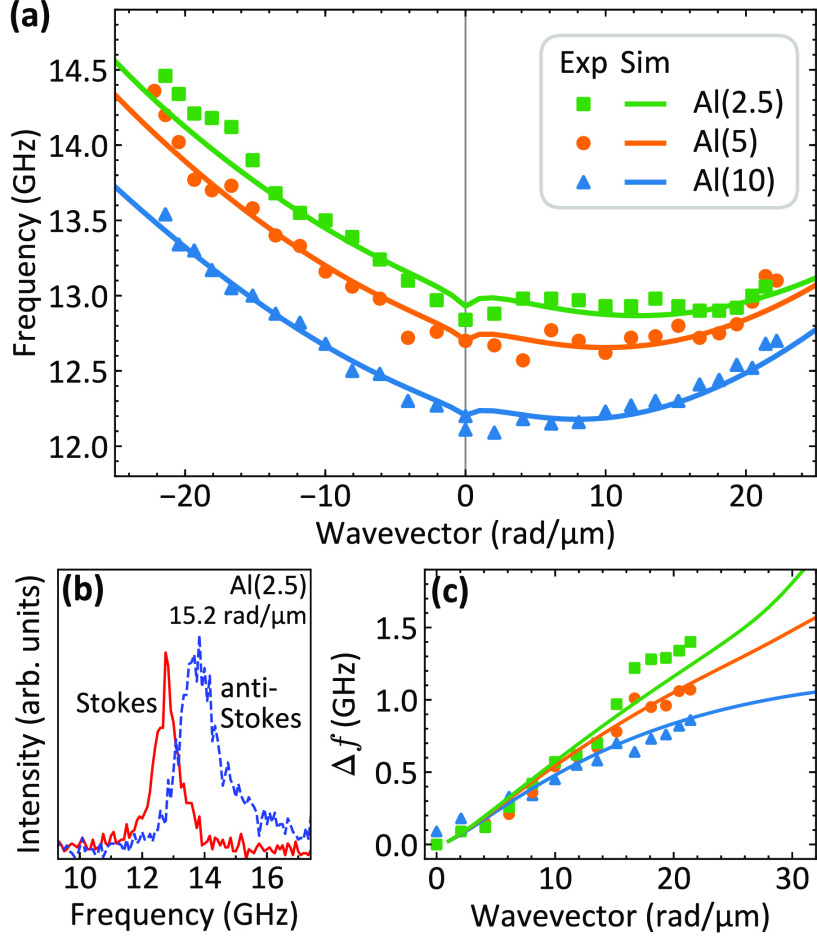
(a) Measured (points) and calculated (lines) SW dispersion relations in the Damon-Eshbach configuration for three samples with different thicknesses of the Al spacer, saturated by an external field *H* = 200 mT. Points at positive (negative) wavevectors are the frequencies collected from the Stokes (anti-Stokes) peaks in the BLS spectra. (b) Absolute frequency of the Stokes and anti-Stokes peaks in BLS spectra measured at *k* = 15.2 rad/μm for the Al(2.5) sample. (c) Frequency difference Δ*f* between anti-Stokes and Stokes peaks as a function of wavevector *k*.

Spin-wave propagation in the trilayer samples has been investigated by the BLS technique. First, the SW dispersion relation has been measured when both the Py and NdCo_7.5_ films are saturated with an in-plane magnetic field *H* = +200 mT along the in-plane easy direction (*x*-axis). BLS measurements (see the Experimental Section for details) have been performed in the Damon-Eshbach configuration, sweeping the in-plane transferred wavevector *k* along the perpendicular direction (*y*-axis). Figure 3a shows the measured frequencies (points) of the Stokes peaks (which arise from SWs propagating with positive *k*) and the anti-Stokes peaks (which arise from SWs propagating with negative *k*) as a function of the SW wavevector. As can be seen, the two peaks exhibit different dispersion relations, and the mode propagating with −*k* is characterized by a larger gradient. This is clearly visible in the BLS spectra shown in Figure 3b, where we observe a significant frequency asymmetry between the Stokes and the anti-Stokes peaks. Such a frequency difference between Damon-Eshbach modes propagating in opposite directions is well reproduced by theoretical calculations (lines) and can be ascribed to the dipolar interaction between the Py and NdCo layers produced by the dynamic components of the magnetization.^49^ A similar frequency difference is present in the calculated dispersion of the modes localized in the NdCo layer. The absence of these modes in the experimental BLS spectra can be explained taking into account that the signal from NdCo layer is much weaker than from Py due to both the finite penetration depth of the laser light in the BLS measurements and the high damping constant of NdCo that leads to a broadening of the BLS peaks. Figure 3c shows the measured (points) and the calculated (lines) frequency difference Δ*f* between the Stokes and the anti-Stokes peak as a function of the wavevector. When the Al thickness is reduced, the increase of the dipolar coupling causes an increase of the frequency asymmetry. For the sample with a 2.5 nm-thick Al spacer, Δ*f* increases almost linearly as a function of wavevector, reaching a value of about 1.4 GHz at *k* = 21.4 rad/μm. Increasing the thickness of the Al spacer, Δ*f* assumes lower values and its slope is reduced for large wavevectors. This effect stems from the decreasing range of the dynamic stray field for large wavevectors, resulting from a weaker dynamic dipolar interaction,^49^ and thus in reaching the maximum value of the nonreciprocity at relatively short wavelengths.

Interestingly, a significant increase of the measured SW frequencies with decreasing the Al spacer thickness can be observed. However, for saturated Py and NdCo layers, interacting only via dipolar coupling, the SW frequency at *k* = 0 is not expected to be affected by the thickness of the Al spacer.^49^ Therefore, the above-mentioned behavior can be ascribed to a reduction of the effective magnetization of the Py film due to the increase of the surface roughness when the thickness of the Al spacer increases. This is confirmed by X-ray reflectivity measurements of Si/Al/Py samples with different thickness of the Al layer. A progressive deterioration of the Al/Py interface with increasing the Al thickness, due to the increase of the roughness of the Al layer, is observed.

### Spin-Wave Dynamics at Remanence

**Figure 4:**
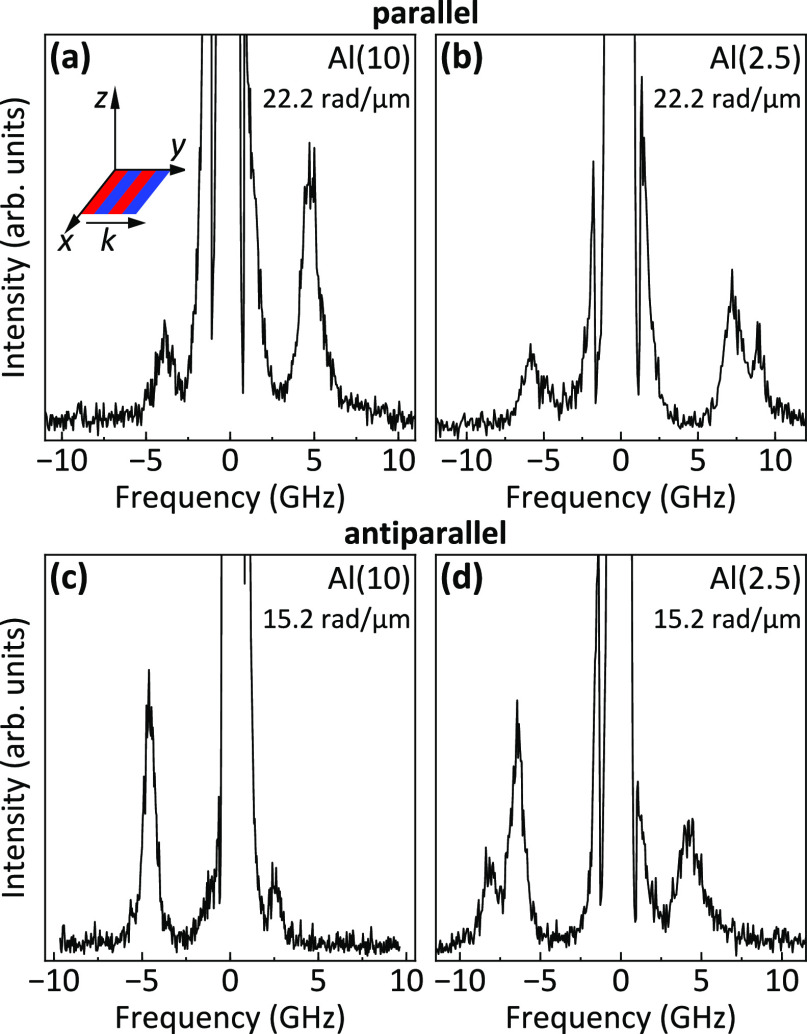
BLS spectra measured at *k* = 22.2 rad/μm and *k* = 15.2 rad/μm for parallel state (top row) and antiparallel state (bottom row), respectively, for the samples with a (a, c) 10 nm-thick and (b, d) 2.5 nm-thick Al spacer. The numbers in parentheses denote the thickness of the Al spacer in nm.

As a second step of our BLS investigation, the SW dispersion has been measured at remanence, when the stripe domains are aligned along the in-plane easy direction (*x*-axis). Also, in this case, the in-plane wavevector has been swept along the *y*-axis, i.e., in the direction perpendicular to the axis of the stripe domains. Figure 4 shows typical BLS spectra recorded in the parallel state (top row) and the antiparallel state (bottom row). For the samples with the 10 nm-thick and 5 nm-thick Al spacer, only one peak, characterized by a sizable frequency asymmetry between the Stokes and the anti-Stokes side, is observed in the BLS spectra. For the sample with the 2.5 nm-thick Al spacer, a second peak is present at higher frequencies in the anti-Stokes (Stokes) part of the BLS spectra for parallel (antiparallel) state for wavevectors larger than about 15 rad/μm.

**Figure 5:**
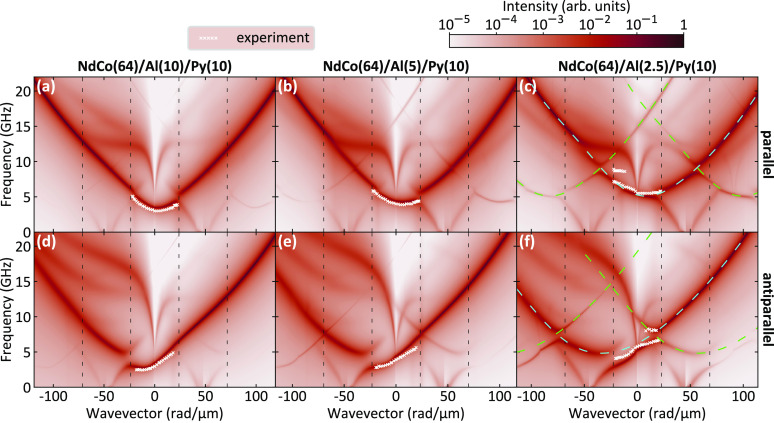
Measured (white crosses) and calculated (color map) dispersion relations for the three investigated samples in two magnetic configurations: (a–c) parallel and (d–f) antiparallel. The color map represents the intensity of the SWs in the Py layer in logarithmic scale obtained from numerical simulations taking into account only the perpendicular dynamic magnetization component (*m*_*z*_). Vertical dashed black lines mark the Brillouin-zone boundaries. Dashed cyan and green lines depict the approximate shape of a parabolic fundamental mode in Py.

The comparison between the calculated (color map) and the measured (white crosses) SW dispersion at remanence is reported in Figure 5 for all of the investigated samples. Since the dynamic magnetization component perpendicular to the film surface gives the main contribution to the BLS cross-section, only the magnetization component *m*_*z*_ was taken into account for the SW intensity calculated using eq 5 (see Experimental Section) and presented with a color map in logarithmic scale. The calculated band structures are very feature-rich and characterized by a marked influence of the periodic pattern of the stripe domains in both the parallel and the antiparallel states. In particular, the band diagram is characterized by a significant frequency asymmetry induced by the dipolar coupling, and this is confirmed by the measured BLS data that correspond to the most intense peak in the calculated dispersion relation. Note that only the first BZ is accessible by BLS, because the maximum wavevector value achievable by this technique is limited to about 22 rad/μm. Interestingly, the bands are present only every second BZ (e.g., the parabolic highest-intensity mode marked with cyan and green lines in Figure 5c,f) as if the period of the domain structure is two times smaller. Indeed, if one considers a period being a single-domain width, the *m*_*x*_ component is periodic while *m*_*y*_ and *m*_*z*_ components are antiperiodic. We have calculated the dispersion relation considering the contribution to the intensity from other dynamic magnetization components. Interestingly, the antiperiodic *m*_*y*_ and *m*_*z*_ components give similar results while the dispersion relation for the periodic *m*_*x*_ component is shifted by the reciprocal space vector along the wavevector axis. We found that this effect is indeed the result of periodicity of magnetization components combined with the conservation of the spin precession direction in the stripe-domain structure. A detailed analysis of each magnetization components is presented in the Supporting Information.

**Figure 6:**
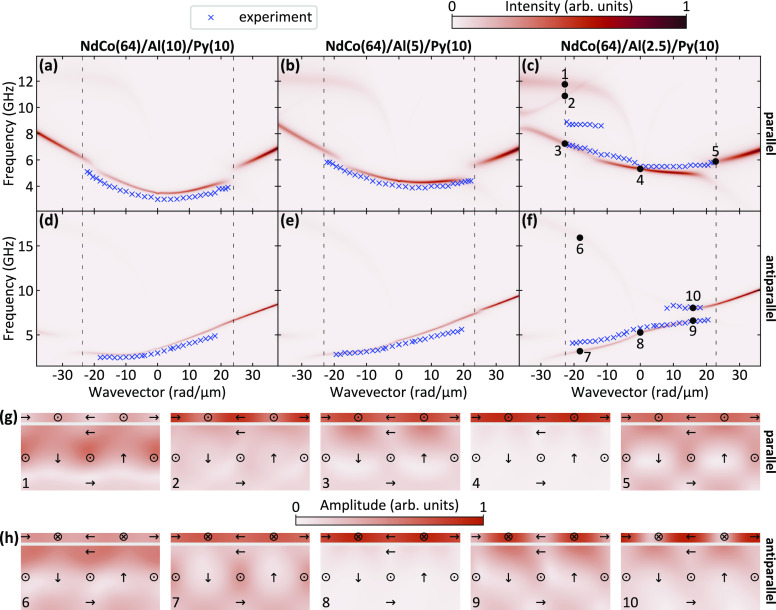
Top panels: dispersion relations of the three investigated samples in two magnetic configurations: (a–c) parallel and (d–f) antiparallel. The color map represents the intensity of the SWs in the Py layer in linear scale obtained with numerical simulations and blue crosses indicate the experimental results. Bottom panels: amplitude of the selected SW modes of the structure with 2.5 nm-thick Al layer in (g) parallel and (h) antiparallel state. Modes are marked with black circles on the dispersion relation plots (c) and (f). The numbers near black circles correspond to the numbers in the bottom-left corners of the sketches. The arrows in the mode amplitude pictures represent the static configuration of the magnetization.

In Figure 6, we present a more-detailed comparison between the calculated band structure (this time in linear scale) and the experimental points, in a restricted range of wavevector values. It can be seen that for the sample with a 2.5 nm-thick Al spacer, the formation of two stationary modes with a band gap having a width of about 1.6 GHz has been experimentally observed near the boundary of the first BZ. Moreover, it is important to note that the opening of the band gap occurs only on one side of the BZ, depending on the magnetic configuration of the system. Specifically, it appears at negative wavevectors for the parallel state and at positive wavevectors for the antiparallel one. This behavior can be explained by taking into account that the band-gap formation is caused by the hybridization of the highest-intensity mode (marked with a dashed cyan line in Figure 5c,f) and the folded branches (marked with a dashed green lines) induced by the stripe-domain periodicity. Due to the strong frequency asymmetry of the band structure, in the parallel (antiparallel) state, the mode hybridization is shifted from the edge of the BZ and occurs only for negative (positive) wavevectors, resulting in an asymmetrical opening of the frequency gap. One can see that in the antiparallel state, the numerical simulations reproduce very well the opening of the experimentally observed band gap, while a small discrepancy between the experimental results and the calculated band diagram is found for the parallel state. The lack of quantitative agreement can be ascribed to the fact that the numerical simulations have been performed using the same values of the NdCo magnetic parameters for all of the investigated samples.

We further note that the highest-intensity mode hybridizes only with a few modes, despite crossing many other modes. This behavior can be ascribed to the high damping of the NdCo layer that does not only affect the intensity of modes localized in the NdCo layer but also causes the hybridizations to diminish^52^ (see Supporting Information).

We further illustrate the characteristics of the calculated modes, see Figure 6g and h, where the spatial distribution of the SW amplitude of selected modes, for both parallel and antiparallel states, is presented for the sample with the 2.5 nm-thick Al spacer. At *k* = 0, the mode having the largest intensity is mainly localized in the Py layer and exhibits an almost uniform spatial distribution for both the parallel (point 4) and the antiparallel (point 8) states. However, with increasing wavevector, this mode couples more strongly to the NdCo layer, and it exhibits a positive dispersion for both positive and negative wavevectors. Moreover, it is characterized by a sizable frequency asymmetry, with a larger slope in the negative wavevectors range, due to dynamic dipolar coupling between the Py and NdCo layers. On the other hand, in the antiparallel state, this mode shows a positive (negative) dispersion in the positive (negative) wavevector range, due to the reversal of the magnetization component *m*_*x*_ in the Py layer, where the mode is mainly localized. We further note that upon reducing the thickness of the Al layer, this mode shows a frequency increase in both the parallel and antiparallel state, caused by the stronger influence of the stray field produced by the NdCo film on the Py layer. Furthermore, we analyze the character of the modes involved in the band-gap formation at the boundary of the first BZ. In both the parallel and the antiparallel state, the two stationary modes have different spatial localizations in the Py layer: the mode at higher frequency has its maximum amplitude in the area where the local magnetization is directed along the *y*-axis (points 2 and 10), while the mode at lower frequency is mainly localized in the region magnetized along the *x*-axis (points 3 and 9). On the opposite side of the BZ with respect to the band gap (points 5 and 7), the calculated amplitude of the modes is almost uniform in the Py layer. To complete the analysis of the modes, we observe a highly dispersive mode which is present for negative wavevectors in both the parallel and antiparallel state for all of the investigated samples. This mode is localized in the area magnetized along the *x*-axis in both the NdCo and the Py layers and has a maximum amplitude at the top surface of NdCo (points 1 and 6). Such a spatial localization of intensity and its strongly dispersive character indicate that this mode originates from the Damon-Eshbach surface mode in the NdCo film. Its absence in the BLS spectra can be ascribed to the high damping in the NdCo layer, as discussed in detail in the Supporting Information.

## Conclusions

Detailed experimental and numerical investigations of the magnetization texture and SW dynamics in NdCo_7.5_(64 nm)/Al(2.5, 5, 10)/Py(10) trilayers have been performed and their usefulness for magnonic applications was discussed. In this system, the hard magnet with PMA develops a stable stripe-domain structure at remanence, which imprints the magnetization stripe texture onto a dipolar-coupled soft magnetic thin film. Two stable configurations, corresponding to the parallel and antiparallel alignment of the Py and NdCo magnetization component along the stripe domains axis, can be achieved at remanence by minor-loop switching at a small magnetic bias field of the order of 20 mT. BLS measurements have shown a marked influence of the imprinted magnetization texture on the dispersion relation of SWs in the Py film. A strongly asymmetric dispersion relation has been found in both configurations, though with opposite asymmetry. Moreover, in the sample with the thinnest Al layer, where the influence of the stray field produced by the NdCo film on the Py layer is more pronounced, the opening of a band gap at the boundary of the first BZ has been experimentally observed. The hybridization measured by BLS in the parallel and antiparallel state was identified as a complex process involving the Py film SW band folding from the second BZ. This demonstrates the formation of a magnonic band structure in the Py film and thus represents a magnonic crystal in the homogeneous Py film by imprinting the magnetization texture from the NdCo layer. It is noteworthy that the band-gap opening occurs only on one side of the first BZ due to the nonreciprocal SW band structure. Such results can find applications in reconfigurable and nonreciprocal magnonic devices based on complex magnetization textures.

## Experimental Section

### Sample Preparation

The NdCo/Al/Py trilayer samples were deposited by magnetron sputtering on thermally oxidized Si wafers. Between the Si/SiO_2_ substrate and the NdCo_7.5_ layer, a 5 nm-thick Al film was deposited. The deposition rate of Co, Nd, Py, and Al was 0.72, 0.6, 1.14, and 0.44 Å/s, respectively. Py, Nd, and Al (Co) were deposited at an angle of incidence of 36° (0°) with respect of the substrate normal, under a 10^–3^ mbar Ar pressure.

### Brillouin Light Scattering Spectroscopy

BLS experiments from thermally excited SWs were performed in the backscattering configuration by focusing a monochromatic laser beam of wavelength λ = 532 nm on the sample surface through a camera objective of numerical aperture NA = 0.24. The scattered light was frequency analyzed by a Sandercock-type (3 + 3)-tandem Fabry–Perot interferometer. The SW dispersion was measured at a fixed magnetic field *H* = 200 mT and at remanence by sweeping the wavevector in the range between 0 and 22.2 rad/μm.

### Numerical Simulations

The SW dynamics are described by the Landau-Lifshitz-Gilbert equation:
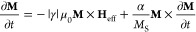
1where **M** = (*m*_*x*_, *m*_*y*_, *m*_*z*_) is the magnetization vector, γ is the gyromagnetic ratio, μ_0_ is the magnetic permeability of vacuum, **H**_eff_ is the effective magnetic field, α is the damping constant, and *M*_S_ is the saturation magnetization. The effective magnetic field **H**_eff_ is described as follows:
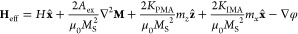
2where *H* is the external magnetic field, *A*_ex_ is the exchange stiffness constant, *K*_PMA_ is the perpendicular magnetic anisotropy constant, *K*_IMA_ is the in-plane magnetic anisotropy constant, and φ is the scalar magnetostatic potential, which can be determined from the magnetostatic Maxwell equations in the form of Poisson-like equation:
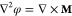
3The finite-element method simulations were performed using COMSOL Multiphysics. A 2D model was used assuming an infinite uniform structure along the *x*-axis. The structure in the saturated state was analyzed with the 2D Landau-Lifshitz-Gilbert equation using the linear approximation of eq 1 assuming *m*_*y*_, *m*_*z*_ ≪ *m*_*x*_ ≈ *M*_S_.^51^ The dispersion relation was calculated by numerically solving the eigenproblem of eqs 1 and 3 for each wavevector separately.^53^ The structure in the parallel and antiparallel states was analyzed using the full 3D Landau-Lifshitz-Gilbert equation. First, time-domain simulations were used to relax a single unit cell with the predefined domain structure [ *m*_*x*_ = *M*_S_|*cos*(2*πy*/*a*)|, *m*_*y*_ = *M*_S_ sin(2*πy*/*a*) in the NdCo layer, *m*_*x*_ = +(−)*M*_S_ in the Py layer in parallel (antiparallel) state] as a function of the lattice constant *a* to find the configuration of the minimum energy. In the second stage, eigenproblem was solved to calculate the dispersion relation, analogously to the saturated state.

The expected BLS intensity of the *n*th SW mode of the frequency *f*_*n*_ is calculated as
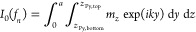
4where *z*_Py,bottom_ and *z*_Py,top_ are the positions of the bottom and top interface at the Py layer. Then every value is turned into the BLS-like peak using the Lorentzian function:
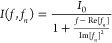
5At the end, the intensities from every mode obtained in the simulations are summed up to create a 2D array of the intensities at any given wavevector and frequency, all of which are then converted into colormaps. The intensity is calculated only in the Py layer to reproduce the effect of small penetration depth of the laser light in the BLS measurements. The SW amplitude associated with a particular mode with frequency *f*_*i*_ and wavenumber *k*_*i*_ is estimated as
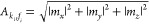
6For the sake of validation, the results from frequency-domain finite-element method simulations were crosschecked with results from micromagnetic simulations in MuMax3. Overall, a good quantitative agreement between these two methods was obtained. However, frequency-domain finite-element method simulations were significantly faster and provided much clearer spectra.

### Material Parameters

The magnetic parameters of Py (reported in Table 1) have been estimated from BLS measurements in the saturated state. The effective magnetization *M*_eff_ = *M*_S_ – 2*K*_PMA_/μ_0_*M*_*S*_ (resulting from the competition between the shape anisotropy energy and the perpendicular anisotropy field), the in-plane uniaxial anisotropy constant *K*_IMA_, and the effective gyromagnetic ratio γ have been estimated from the measurements of the SW frequency as a function of both the intensity and direction of a saturating in-plane magnetic field, while the exchange constant *A*_ex_ has been estimated from the measurement of the dispersion relation. *M*_eff_ and *A*_ex_ (*K*_IMA_) are found to decrease (increase) upon increasing the Al thickness. This behavior can be ascribed to the increase of the surface roughness when the thickness of the Al spacer increases. The gyromagnetic ratio γ, instead, is independent of the Al thickness and equals 1.85 × 10^11^ rad/s/T. The Gilbert damping was fixed to the value of α = 0.01.

**Table 1:** Magnetic Parameters of Py Layer in the Samples Investigated in the Paper

structure	*M*_eff_ (kA/m)	*A*_ex_ (pJ/m)	*K*_IMA_ (kJ/m^3^)
NdCo(64)/Al(10)/Py(10)	465	7	3.5
NdCo(64)/Al(5)/Py(10)	525	9	3.5
NdCo(64)/Al(2.5)/Py(10)	590	10	1.2

The magnetic parameters of NdCo_7.5_ were determined from both the simulation of the static magnetic configuration and the dispersion relations in the parallel and antiparallel states: *M*_S_ = 1100 kA/m, *A*_ex_ = 10 pJ/m, *K*_PMA_ = 130 kJ/m^3^, *K*_IMA_ = 10 kJ/m^3^, α = 0.1, and γ = 1.85 × 10^11^ rad/s/T.

## Supporting Information Available

Analysis of the effect of damping on spin-wave dynamics; explanation of wavevector shift in dispersion relations of different magnetization components (PDF)
