# Supplementary material for: Reconfigurable Magnonic Crystals Based on Imprinted Magnetization Textures in Hard and Soft Dipolar-Coupled Bilayers
Source: ACS Nano. 2022 Aug 31;16(9):14168–77. doi: 10.1021/acsnano.2c04256 (PMC9527808; doi:10.1021/acsnano.2c04256)
Supplement: Supplementary file 1 — nn2c04256_si_001.pdf [file nn2c04256_si_001.pdf]

SUPPORTING INFORMATION

Reconfigurable Magnonic Crystals Based on Imprinted Magnetization Textures in Hard and Soft Dipolar-Coupled Bilayers

Krzysztof Szulc[1*†], Silvia Tacchi[2*‡], Aurelio Hierro-Rodríguez[3,4], Javier Díaz[3,4], Paweł Gruszecki[1], Piotr Graczyk[5], Carlos Quirós[3,4], Daniel Markó[6¶], José Ignacio Martín[3,4], María Vélez[3,4], David S. Schmool[6], Giovanni Carlotti[7], Maciej Krawczyk[1], Luis Manuel Álvarez-Prado[3,4]

[1] *Institute of Spintronics and Quantum Information, Faculty of Physics, Adam Mickiewicz University, Poznań, Uniwersytetu Poznańskiego 2, 61-614 Poznań, Poland*
[2] *Istituto Officina dei Materiali del CNR (CNR-IOM), Sede Secondaria di Perugia, c/o Dipartimento di Fisica e Geologia, Università di Perugia, I-06123 Perugia, Italy*
[3] *Departamento de Fisica, Facultad de Ciencias, Universidad de Oviedo, C/Federico Garcıa Lorca nº 18, 33007 Oviedo, Spain*
[4] *Centro de Investigacion en Nanomateriales y Nanotecnología (CINN), CSIC-Universidad de Oviedo, 33940 El Entrego, Spain*
[5] *Institute of Molecular Physics, Polish Academy of Sciences, M. Smoluchowskiego 17, 60-179 Poznań, Poland*
[6] *Université Paris-Saclay, UVSQ, CNRS, GEMaC, 78000 Versailles, France*
[7] *Dipartimento di Fisica e Geologia, Università di Perugia, I-06123 Perugia, Italy*
[*] *The authors contribute equally to this work*
[†] krzysztof.szulc@amu.edu.pl
[‡] tacchi@iom.cnr.it
[¶] *Current address : Silicon Austria Labs GmbH, Magnetic Microsystem Technologies, Europastraße 12, 9524 Villach, Austria*

Effect of damping in NdCo on spin-wave dynamics in Py

We have investigated the effect of damping in the NdCo layer on the dispersion relation in the Py layer for the parallel and antiparallel states at remanence. The simulations were performed for three values of $\alpha_{\mathrm{NdCo}} = 0.01, 0.1,$ and 0.2. We restrict the analysis to the Al(2.5) sample, but the effects described below can be directly extended to larger Al thicknesses. The results are shown in Figure S1. Lower damping allows the observation of more modes in the dispersion relation. Every mode is approximated by the Lorentzian function described in Equation 5. The imaginary part of the frequency, which describes the full width at half maximum of the peak, depends on the value of the damping. The larger the damping, the broader the peak and, therefore, lower intensity of lines in Figure S1. As the peak of the highest intensity mode is almost independent of the NdCo damping (as it is concentrated in the Py layer), it produces an almost constant ambient intensity. If the damping is large enough, the modes concentrated in the NdCo layer are below this ambient intensity and disappear from the plots. Moreover, the damping has a large impact on the hybridizations between the modes. For $\alpha_{\mathrm{NdCo}} = 0.2$, the mode of the highest intensity has almost no hybridizations except for the hybridization between its corresponding modes from neighboring Brillouin zones (Figure S1c) and hybridization with the low-frequency mode (Figure S1f). When the damping is lower, the hybridizations with the other modes appear and should be detectable in the experiment. Interestingly,

the hybridization in the antiparallel state of the Al(2.5) sample (Figure S1f), which is visible in the experiment, seems to involve also the mode originating from the oscillations of the domain structure in the NdCo layer. It also affects the amplitude of the mode (see Figure 6h plots 9 and 10). However, the hybridization between the Py modes from different Brillouin zones are the only visible hybridizations in the BLS spectra, indicating a large damping constant in the NdCo layer.

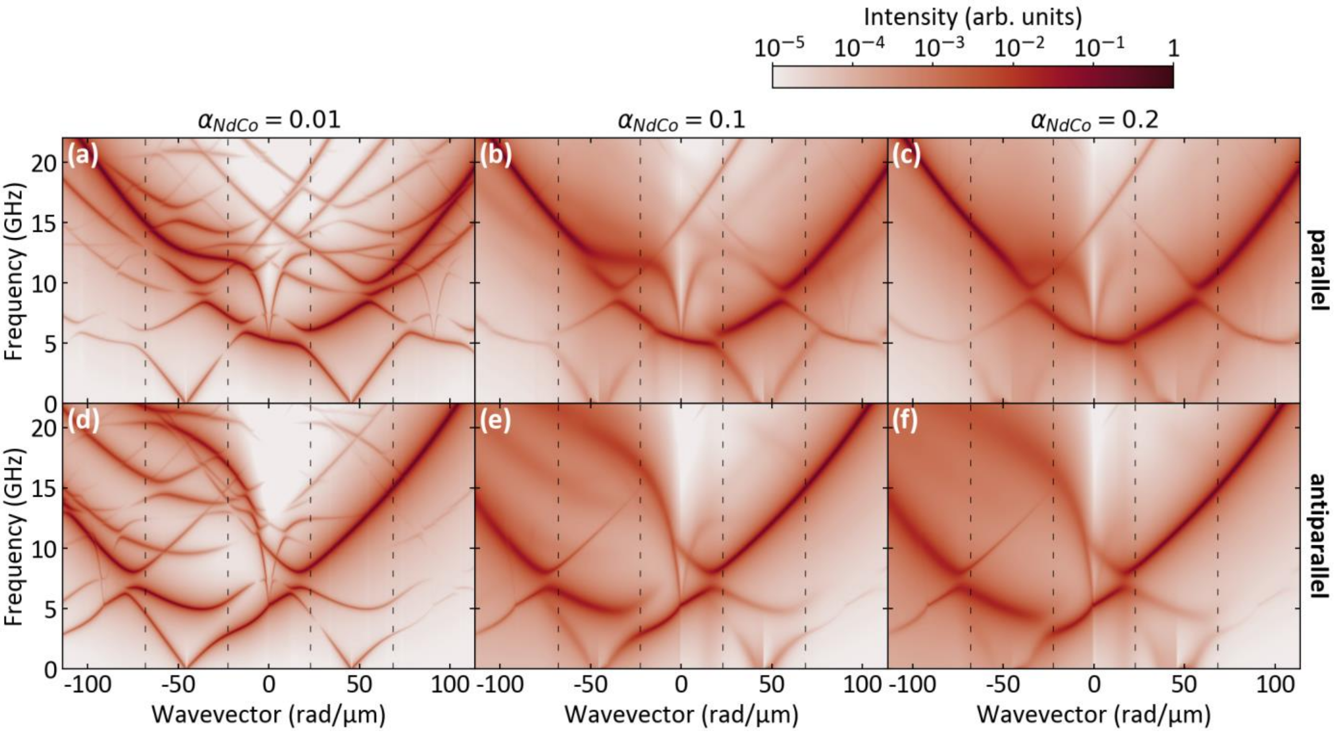

Figure S1. Influence of the damping in the NdCo layer on the dispersion relation of the Py layer in the Al(2.5) sample obtained from numerical simulations.

Double periodicity effect in the spin-wave dynamics in a stripe-domain structure

The dispersion relations for the three components of the dynamic magnetization are shown in Figure S2. The dispersion relations of the m_x and m_y components were calculated by replacing the m_z term in Equation 4 with the m_x and m_y, respectively. As can be seen, the dispersion for m_y and m_z are very similar and exhibit the same features as a function of the wavevector, while in the dispersion of the longitudinal component m_x all the bands are rigidly shifted by a reciprocal-space vector. This behavior has been also found in an investigation of Co/Pd multilayers[1], but its origin was not discussed. In the following, we provide a detailed explanation based on the analysis of the spin precession in adjacent stripe domains.

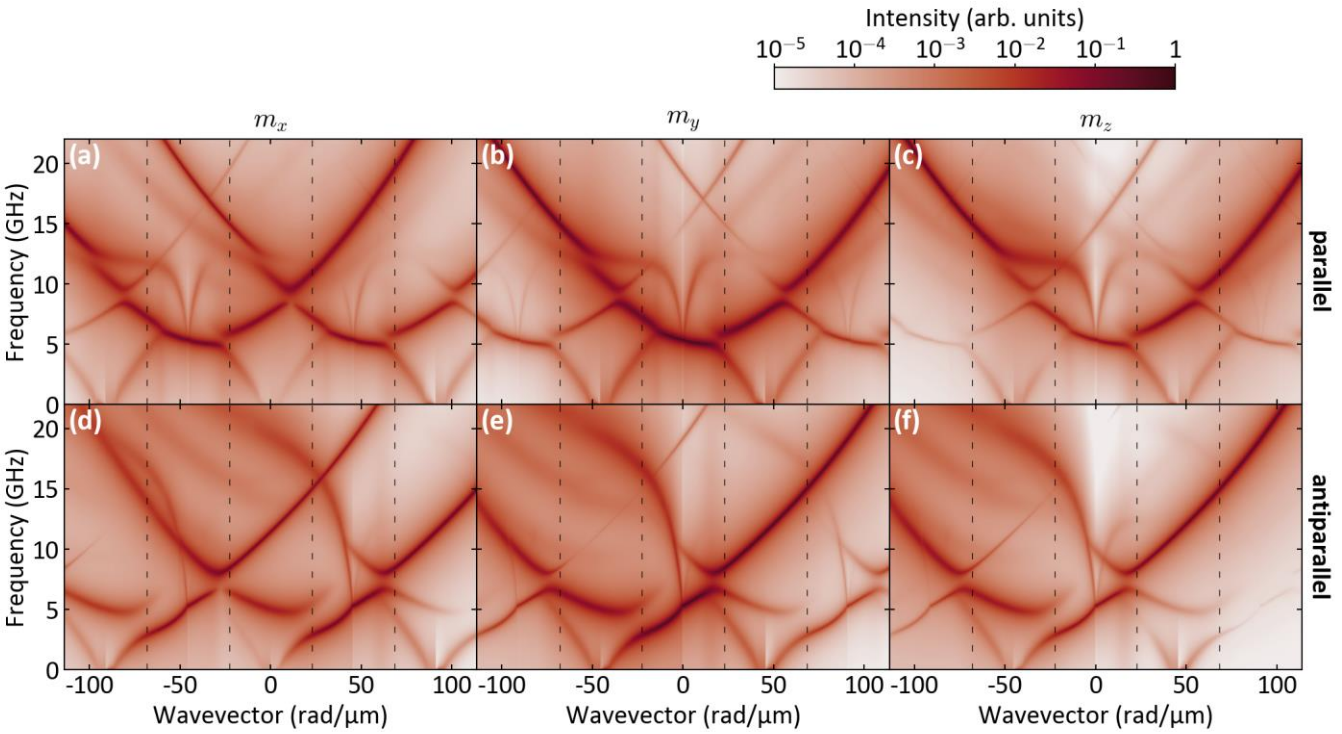

Figure S2. Contribution of the dynamic magnetization components—m_x (left column), m_y (middle column), and m_z [right column, see Figure 5c,f]—to the dispersion relation in the Al(2.5) sample in the parallel (a-c) and antiparallel (d-f) state.

Figure S3 shows a basic model of the stripe domain structure where the magnetization component perpendicular to the film plane is zero: the local magnetization is parallel to the x-axis in the center of the domain walls, while it is directed in the $\pm y$-direction between the domain walls. This structure is analogous to the one imprinted onto the Py layer (see Figure 2), that gives the main contribution to the measured band structure as discussed in the main text, but the magnetization rotates by exactly 90°. Let us now analyze the spin dynamics of the uniform mode with $k = 0$ along the y-axis (perpendicular to the stripe domains) in a single period of the stripe domain pattern visible in top-left corner of Figure S3. The spin-precession direction is counter-clockwise as defined by the Landau-Lifshitz-Gilbert equation. At $t = 0$, all of the dynamic components are directed along the $+z$-direction, since the mode is uniform and the static component lies in the xy-plane. At $t = T/4$, where T is the period of the spin wave, the dynamic component rotates by 90°.

[1] Banerjee, C.; Gruszecki, P.; Klos, J. W.; Hellwig, O.; Krawczyk, M.; Barman, A. Magnonic band structure in a Co/Pd stripe domain system investigated by Brillouin light scattering and micromagnetic simulations. *Phys. Rev. B* **2017**, *96*, 024421.

The spins aligned in the $+x$-direction have their dynamic component directed in the $-y$-direction, while the spins directed in the $\pm y$-direction have their dynamic component directed in the $\pm x$-direction. Similarly, at $t = 3T/4$, the spins directed in the $\pm y$-direction have their dynamic component directed in the $\mp x$-direction, while the spins aligned along the $+x$-direction have their dynamic component directed in the $+y$-direction. It implies that the m_y and m_z components oscillate in-phase (hence correspond to $k = 0$), whereas, the m_x component is shifted by 180° in adjacent domains and, therefore, behaves like a standing wave of wavenumber $k = 2\pi/a$.

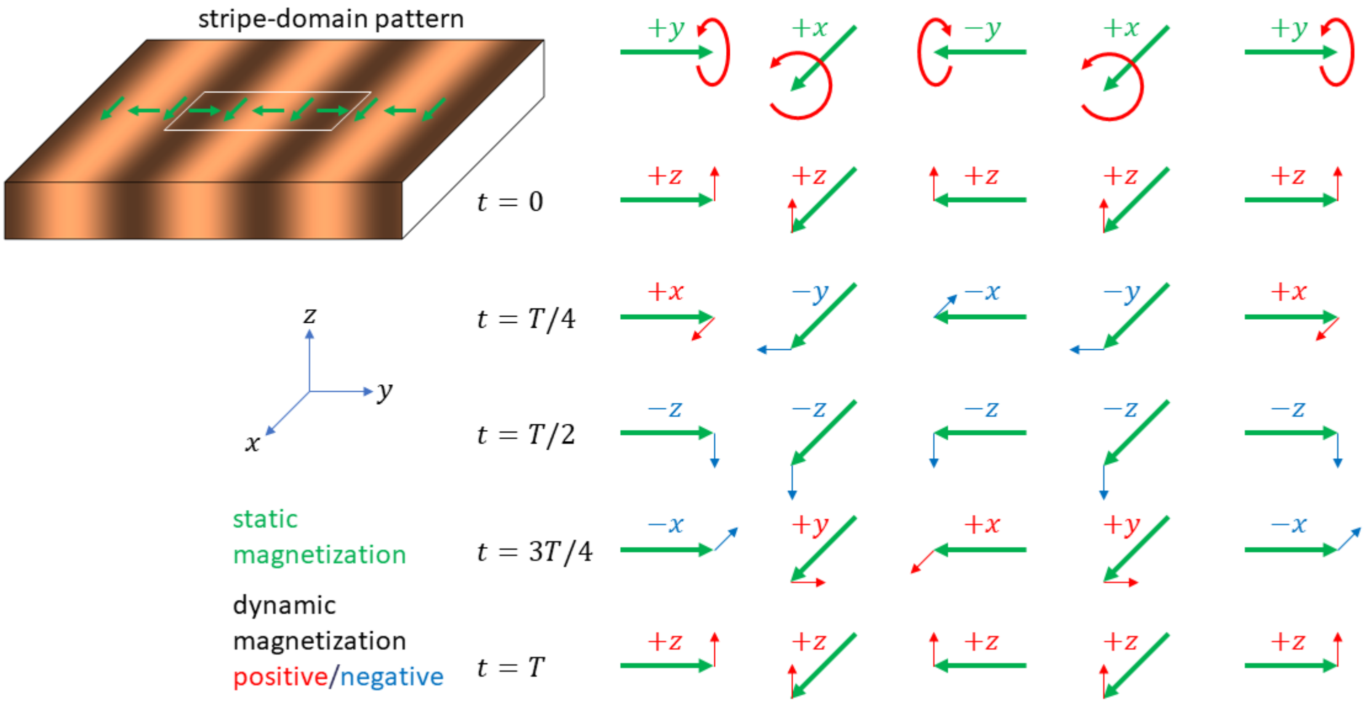

Figure S3. Magnetization dynamics of the fundamental mode at $k = 0$ in a direction perpendicular to the stripe domains (area marked by the white rectangle in the sketch in the top-left corner).
